# Artificial Intelligence in Prenatal Ultrasound Diagnosis

**DOI:** 10.3389/fmed.2021.729978

**Published:** 2021-12-16

**Authors:** Fujiao He, Yaqin Wang, Yun Xiu, Yixin Zhang, Lizhu Chen

**Affiliations:** Department of Ultrasound, Shengjing Hospital of China Medical University, Shenyang, China

**Keywords:** ultrasound, artificial intelligence, prenatal diagnosis, fetus, medical imaging

## Abstract

The application of artificial intelligence (AI) technology to medical imaging has resulted in great breakthroughs. Given the unique position of ultrasound (US) in prenatal screening, the research on AI in prenatal US has practical significance with its application to prenatal US diagnosis improving work efficiency, providing quantitative assessments, standardizing measurements, improving diagnostic accuracy, and automating image quality control. This review provides an overview of recent studies that have applied AI technology to prenatal US diagnosis and explains the challenges encountered in these applications.

## Introduction

Ultrasonography is convenient, low-cost, real-time, and non-invasive, and it has been the most largely used imaging modality. Antenatal ultrasound (US) examination, as the most important imaging method used during pregnancy, can assess the growth condition and birth defects of a fetus, helping the fetus to receive timely and effective treatment before or after delivery. For malformations with a poor prognosis, timely termination of a pregnancy could reduce the rate of births with severe birth defects. However, this time-consuming process depends to a large extent on doctor's experience and the available equipment. Moreover, it involves great work intensity in practice.

Artificial intelligence (AI) (1) refers to the ability to interpret external data and learning for specific purposes through flexible adaptation. Machine learning (ML), a field gaining considerable attention in AI, is a powerful set of computational tools that trains models on descriptive patterns obtained from human inference rules. However, a major problem facing ML is that feature selection relies heavily on statistical insights and domain knowledge, a limitation that initiated the development of deep learning. As a branch of ML, deep learning takes advantage of convolutional neural networks, one of the most powerful methods associated with images, which can realize high performance with limited training samples and even permit more abstract feature definitions. Consequently, it is often used for image pattern recognition and classification.

**Figure 1:**
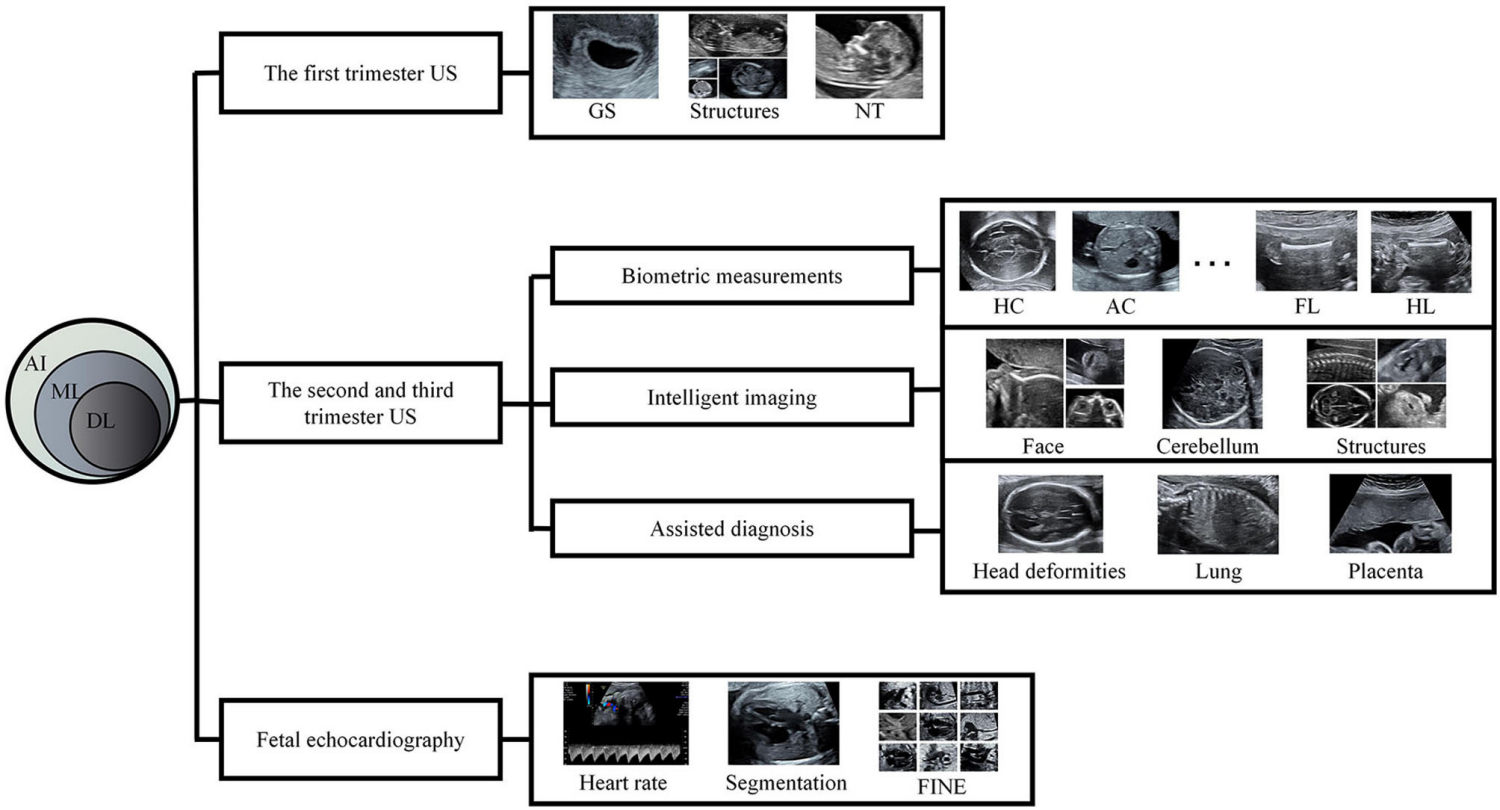
A schematic diagram of this review AI, artificial intelligence; ML, machine learning; DL, deep learning; US, ultrasound; GS, gestational sac; NT, nuchal translucency; HC, head circumference; AC, abdominal circumference; FL, femur length; HL, humerus length; FINE, fetal intelligent navigation echocardiography.

There has been much research on radiology with AI (2–5), and AI-assisted diagnosis has also become a research hotspot in the US field. Some experts have gained success in the intelligent US diagnosis of liver, thyroid, and breast diseases (6–9). However, AI in prenatal US diagnosis is still in its infancy, though there have been breakthroughs in measurement, imaging, and diagnosis. Such applications are of great significance; not only they improve efficiency, but they also make up for the inexperience and skill deficiency of some examiners. In this review, we introduce recent literature on the application of AI in prenatal US diagnosis (Figure 1).

## Ai In The First Trimester Us

### Gestational Sac

GS is the first important structure observed by US in pregnancy. The mean gestational sac diameter can roughly estimate the gestational age (GA). Zhang et al. (10) designed an automatic solution to select the standardized biometric plane of the GS and perform measurements during routine US examinations. The quantitative and qualitative analysis results showed the robustness, efficiency, and accuracy of the proposed method. Although this study is restricted to normal gestation within 7 weeks, it is expected to facilitate the clinical workflow with further extension and validation. Yang et al. (11) established a fully automatic framework that could simultaneously affect the semantic segmentation of multiple anatomical structures including the fetus, GS, and placenta in prenatal volumetric US. Extensively verified on large in-house datasets, the method demonstrates superior segmentation results, good agreement with expert measurements, and high consistency against scanning variations.

### Fetal Biometry Assessment

The automation of image-based assessments of fetal anatomies in the initial trimester remains a rarely studied and arduous challenge. Ryou et al. (12) developed an intelligent image analysis method to visualize the key fetal anatomy and automate biometry in the first trimester. With this approach, all sonographers needed to do was acquire a three-dimensional US (3DUS) scan following a simple standard acquisition guideline. Next, the method could perform semantic segmentation of the whole fetus and extract the biometric planes of the head, abdomen, and limbs for anatomical assessment. However, it exhibited relatively low qualitative analysis results of the limbs due to a low detection rate.

### Nuchal Translucency

NT is a fluid-filled region under the skin of the posterior neck of a fetus, which appears sonographically as an anechogenic area. A fetus with increased NT thickness has a higher risk of congenital heart disease, chromosomal abnormalities, and intrauterine fetal death. The NT measurement has proven to be a crucial parameter in prenatal screening.

NT thickness needs to be measured in the fetal standard medium sagittal plane. On account of the low signal-to-noise ratio of ultrasonic data, the relatively short fetal crown-rump lengths and activity in early pregnancy, the section is hard to obtain, making its intelligent measurement a challenge. In recent years, with the joint efforts of multidisciplinary experts, many breakthroughs have been made (Table 1).

**Table 1:** Summary of studies about intelligent measurements of NT.

**References**	**Technologies**	**Tasks**	**Performances**
Lee et al. (13)	Coherence-enhancing diffusion filter, Dynamic programming	Automatic measurement of NT with manual ROI	Correlation c_a,m_: 0.99
Catanzariti et al. (14)	Dynamic programming	Automatic measurement of NT with manual ROI	No quantitative analysis and perform better than Lee et al. (13)
Deng et al. (15)	SVM classifier, Gaussian pyramids	Automatic detection of the NT region in the standard mid-sagittal plane	Accuracy: 93.1%
Park et al. (16)	Dijkstra's shortest path, Oriented gradient filters, Graph Cut segmentation, Hierarchical Detection Network	Automatic segmentation and measurement of NT in the standard mid-sagittal plane	The detection results are accurate for most cases
Siqing et al. (17)	Dynamic programming, Hessian plate filter, Deep belief network	Automatic identification the mid-sagittal plane, detection, and measurement of NT	σ = 0.40, d_NTlen_ = 0.28, d_border_ = 0.27
Sciortino et al. (18)	Wavelet, Multi resolution analysis	Automatic identification the mid-sagittal plane, detection, and measurement of NT	Sensitivity: 99.95%

*NT, nuchal translucency; ROI, region of interest; Correlation c_a,m_, defined as correlation between semi-automatic and manual measurements for the maximum NT thickness; SVM, support vector machine; σ, the variance of NT thickness among the dataset; d_NTlen_ is the error of the automatic measurement of NT thickness comparing with the manual measurement; d_border_ is the average error of the automatic detected borders and manually drawn ones*.

Initially, the thickness of NT could be measured by manual selection of the region of interest (13, 14), and semi-automatic approaches were proven to produce reliable measurements compared to traditional manual methods. Later, experts made attempts to automatically identify and measure NT in mid-sagittal section images (15, 16), and the NT detection results were accurate in most cases. Following studies of the automatic detection of the fetal sagittal plane in US (19, 20), researchers developed sufficiently accurate (17, 18) methods for the automatic recognition of the standard NT plane as well as measurements of its thickness.

## Ai In The Second And Third Trimester Us

It is in the mid-trimester that sonographers can evaluate fetal growth and find dysplasia with greater sensitivity. The second trimester scan associated with third trimester checks can better detect congenital abnormalities and even predict postnatal outcomes.

### Biometric Measurement

Standardized measurements are an indispensable part of prenatal US, which play a role in dating pregnancies and detecting potential abnormalities, but the process remains a highly repetitive one. Automation assists in reducing the time needed for routine tasks, allowing more time to analyze additional scan planes for diagnosis. Moreover, automatic measurements can reduce operator bias and contribute to improved quality control.

#### Fetal Head

A number of AI-based methods have been developed for head circumference (HC) measurement (21–28), the studies of which are summarized in Table 2.

**Table 2:** Summary of studies about intelligent measurements of HC.

**References**	**Task**	**Data used for learning**	**GA (for training and application)**	**Total number of images (total number of pregnant)**	**Number of testing images**	**Methods**	**Dice**
Foi et al. (21)	Segmentation + Measurement	2D	21, 28, and 33 weeks		90	Nelder - Mead	0.96
Zhang et al. (22)	Detection + Segmentation + Measurement	2D	20–35 weeks	41 (41)	21	Supervised texton + RF	0.97
Li et al. (23)	Detection + Measurement	2D	18–33 weeks	669	145	RF + Ellifit	0.97
Sinclair et al. (24)	Detection + Measurement	2D	18–22 weeks	2,703 (2724)	539	CNN	0.98
Vandenheuvel et al. (25)	Detection + Measurement + Gestation	2D	All trimesters	1,334 (551)	333	CNN + U-net	0.97
Kim et al. (26)	Detection + Measurement + Checking	2D		172	70	U-Net + CNNs + Ellifit	0.95
Sobhaninia et al. (27)	Segmentation + Measurement	2D	All trimesters	999	250	CNN + U-net	0.97
Li et al. (28)	Segmentation + Measurement	2D	All trimesters	1,334 (551)	335	CNN	0.97

*HC, head circumference; GA, gestational age; Dice, a parameter describing the similarity between the performance of proposed method and the ground truth; 2D, two dimensional; RF, random forests; CNN, convolutional neural network*.

Fetal factors—such as abnormalities, low contrast, speckle noise, boundary occlusion, or other artifacts—may affect intelligent detection and measurement; such conditions having been accounted for in the studies (22, 24, 26, 28). Approaches with plane verification may obtain more accurate results. As Kim et al. (26) assessed the transthalamic plane on the basis of the cavum septum pellucidum, the V-shaped ambient cistern, and the cerebellum. Van den Heuvel et al. (25) combined their intelligent method with the Hadlock curve, allowing the GA to be determined automatically from the measurements. However, the determination was unreliable for the third trimester.

Some medical devices have been successfully equipped with software for intelligent processing (29–31). With such computer assistance, basic planes can be extracted and biometries related to the fetal head can be obtained automatically from the 3DUS. And the computer-assisted systems have proven to be reliable in measuring HC. With further optimization, such tools suggest great promise in improving workflow efficiency.

#### Fetal Abdomen

The low contrast between the fetal abdomen and the surrounding environment, its irregular shape, and the high variability of images all serve to make intelligent study of the abdominal circumference (AC) a difficult task. Several original studies (32–34) published on the subject are summarized in Table 3.

**Table 3:** Summary of studies about intelligent measurements of AC.

**References**	**Task**	**Technologies**	**Sample**	**GA of data used**	**Results**
Wang et al. (32)	Detection+Measurement	HT+local phase	590	18–39 weeks	Measurement: MSD 0.42%; SD 2.91%; P value 0.16.
Jang et al. (33)	Classification+Measurement+Checking	CNN+HT	88		Measurement: Dice 0.85; check: accuracy 80.9%.
Kim et al. (34)	Detection+Measurement+Checking	CNN+U-net	174		Measurement: Dice 0.93; check: accuracy 87.1%.

*AC, abdominal circumference; GA, gestational age; HT, Hough transform; MSD, mean sign difference; SD, standard deviation; P value, help to further assess the statistical evidence; CNN, convolutional neural network; Dice, a parameter describing the similarity between the performance of proposed method and the ground truth*.

The novelty of a method introduced by Kim et al. (34) was the use of the spine position as a navigation marker to determine the final plane for AC measurement. In this way, the influence of interference—such as a regional lack of amniotic fluid, acoustic shadows, or certain anatomical structures—could be reduced. Moreover, the checking process of the standard plane further raises the stability and accuracy of AC measurements. This intelligent approach significantly outperforms conventional studies (33), and the multiple learning framework is desirable to integrated into a single framework.

#### Fetal Long Bone

The position and posture of a fetus varies, which limits such studies about fetal long bone. There has been some progress regarding the segmentation and measurement of the fetal femur (22, 35–37) (Table 4). Most of the methods can be divided into several parts including the determination of regions of interest, image processing, identification of femoral features, and measurement of lengths or volumes. Moreover, the models can obtain similar accuracy as that obtained via manual measurements.

**Table 4:** Summary of studies about intelligent measurements of FL.

**References**	**Task**	**Technologies**	**Data used**	**GA of data used**	**Samples**	**Results**
Yaqub et al. (35)	Segmentation	RF	3D	13–25 weeks	51	Segmentation: precision 87.0%; recall 82.0%; Dice 0.83
Hur et al. (36)	Reconstruction + Measurements		3D	26–32 weeks	39	Measurement: successful rates for femur, tibia and fibula length are 96.1, 80.7, and 76.9%
Zhang et al. (22)	Segmentation + Measurement	Supervised texton + RF	2D	20, 21, 28, 34, 35 weeks	30	Measurement: accuracy 99.8%; precision 77.6%; recall 95.2%; specificity 99.8%; Dice 0.86
Luo et al. (37)	Segmentation + Measurement	Frangi filter	2D	18–27 weeks	70	Measurement: precision 57.5%; recall 85.3%; specificity 99.8%; Dice 0.73

*FL, femur length; GA, gestational age; RF, random forests; 2D, two dimensional; 3D, three dimensional; Dice, a parameter describing the similarity between the performance of the proposed method and the ground truth*.

Hur et al. (36) conducted a prospective study to evaluate the performance of a 3DUS system, five-dimensional long bone (5DLB), in detecting the lower limb long bone. They found this intelligent tool to be reproducible and comparable with conventional two-dimensional (2D) and manual 3D techniques for fetal long bone measurements. As the study demonstrates, the new technique streamlines the process of reconstructing lower limb long bone images and performing fetal biometry.

#### Multiple Structures

With the advances in ML, professionals were not satisfied by focusing on a single anatomical structure alone and started exploring measurements of multiple structures. Carneiro et al. (38, 39) proposed a novel method for the rapid detection and measurement of fetal anatomical structures. The system was, on average, close to the accuracy of experts in terms of the segmentation and obstetric measurements of HC, AC, femur length, etc. The approach dealt with humerus length and crown-rump length measurements for the first time. Moreover, the framework was further optimized for an intelligent application—that is, syngo Auto OB measurements algorithm—and demonstrated acceptable performance when integrated with the clinical workflow (40).

### Intelligent Imaging

Accurate acquisition of fetal standard planes with key anatomical structures is crucial for obstetric examination and diagnosis. However, the standard plane acquisition is a labor-intensive task and requires an operator equipped with a thorough knowledge of fetal anatomy. Therefore, automatic methods are in high demand to alleviate the workload and boost examination efficiency.

The fetal face is one of the key points of a prenatal US scan, and conventional 2DUS is still the gold standard for the examination. Automatic solutions (41, 42) for the recognition of the fetal facial standard plane (FFSP) have been presented using different models. They can classify input images into the axial plane, coronal plane, sagittal plane, and non-FFSP. Lei et al. (41) used manual annotated features from consecutive US images to train their intelligent system. In the literature (42), method have been proposed that learn feature representations from raw data for recognition without any manually designed features via deep convolutional neural network architectures. Representations discovered by deep convolutional neural networks are more robust and sophisticated than standard hand-crafted features, facilitating better classified results.

Assessment of the fetal cerebellar volume is important for evaluating fetal growth or diagnosing nervous system deformities. However, the irregular shape of the cerebellum and strong ultrasound image artifacts complicate the task without manual intervention. AI in prenatal US examination has realized the automatic localization (43), and segmentation (44) of the fetal cerebellum in 3DUS with reasonable accuracy.

Owing to the development of computer technology, the emergence of medical AI tools make it possible to automatically detect the genital organ (45) and kidney (46) with an accuracy of more than 80%. Moreover, the intelligent imaging of multiple different fetal structures has also become a reality based on CNNs. Chen et al. (47) presented a general framework for the automatic identification of four fetal standard planes—including the abdominal, face axial, and four-chamber view standard plane—from US videos. Extensive experiments have been conducted to corroborate the efficacy of the framework on the standard plane detection problem. Baumgartner et al. (48) proposed a novel method to automatically detect thirteen fetal standard views including the brain, lips, kidneys, etc. Moreover, it could provide the localization of the target structures via a bounding box. Evidence in the experimental data suggested that the proposed network could achieve excellent results for real-time annotation of 2DUS frames. While Sridar et al. (49) introduced and assessed a method to automatically classify fourteen different fetal structures using 2DUS images. After verification, there was good agreement between the ground truth and the proposed method. The architecture was capable of predicting images without US scanner overlays with a mean accuracy of 92%. Although these studies focused only on pregnant woman of 18–22 weeks GA, they initiated new ideas for prenatal US studies.

### Assisted Diagnosis

Diagnosis of fetal abnormalities in US is a highly subjective process. Consequently, the research into computer-aided diagnosis (CAD) tools will help doctors make more objective and quantitative decisions. However, the ML of abnormal cases is a complex study in itself and needs sufficient training data—currently, there are but a few studies.

#### Central Nervous System Abnormalities of Fetus

Sahli et al. (50) proposed a learning framework for the automatic diagnosis of microcephaly and dolichocephaly using intelligent measurements of fetal head. Test results showed that this method could detect and diagnose such abnormalities quickly and accurately, but it focused only on size and shape, excluding internal structures.

Based on previous experts' research, Xie et al. (51) added specific abnormal cases to the exploration of CAD. The study included a total of 29 419 images from 12,780 pregnancies, containing cases of common central nervous system abnormalities confirmed by follow-up care such as, ventriculomegaly, microcephalus, holoprosencephaly, etc. In addition to recognize and classify normal and abnormal US images of the fetal brain, their method could also visualize lesions and interpret results using heat maps. After testing, the overall accuracy for classification reached 96.31%; the probability of heat map location being 86.27%. This study confirmed the feasibility of the CAD of encephalic abnormities. It also laid the foundation for further study of the diagnosis and differential diagnosis of fetal intracranial malformations. Xie et al. (52) proposed a similar CAD approach for the differential diagnosis of five common fetal brain abnormalities. The algorithms, however, need further refinement for diagnosis assistance and the reduction of false negatives.

#### Fetal Lung Maturation

Neonatal respiratory morbidity (NRM) is the leading cause of mortality and morbidity associated with prematurity, and it can be assessed through the fetal lung maturity (FLM) process. Traditional clinical options for FLM estimation are either the use of GA directly as a proxy FLM estimator or through amniocentesis, an invasive laboratory test. In recent years, the feasibility of evaluating the degree of fetal lung maturation from US images has been preliminarily validated (53, 54). Moreover specialists have made attempts to quantitatively analyze FLM by means of ML (55, 56). Such automated and non-invasive methods can predict NRM with a performance similar to that reported for tests based on amniotic fluid analysis and much greater than that of GA alone. The intelligent evaluation technique needs further studying and is a promising technique to assist clinical diagnosis in the future.

#### Placenta

US classification of placental maturity is a key part of placental function evaluation. At present, determining the placenta stage depends mainly on observation and the empirical analysis of clinicians. The emergence of automatic detection provides an alternative for the evaluation which would reduce the differences in subjective evaluations improving and verifying the diagnoses.

With the development of computer techniques, some intelligent grading methods have been developed (57, 58), but analysis results are insufficiently good for practical application owing to fewer discriminative features, sample methods for classification, etc. The approach of Lin (59) included several algorithms for extracting different characteristics and a process for selecting multiple relevant characteristics. The performances of different methods for feature extraction were compared in the article and confirmed that their method outperformed previous studies, providing more accurate staging results. Consequently, it exhibited better clinical value. In other studies (60, 61), the use of dense sampling contributes to better discriminability as more placental image samples are captured. They share the same classifier and descriptor but have different encoding methods. And Lei et al. (61) achieved obvious superiority in encoding accuracy. Intelligent evaluation methods could be further optimized by adding more advanced algorithms or synthesizing other information such as blood flow.

## Ai In Fetal Echocardiography

Congenital heart disease (CHD) is one of the most common birth defects. Fetal echocardiography, as the preferred choice for diagnostic screening and prognosis evaluation, is getting increased attention. However, this examination is resource limited, and the diagnostic accuracy depends on the skills of the screening operators. According to a multicenter study done in China, despite the specificity of fetal echocardiography being 99.8%, the sensitivity was just 33.9% (62). AI research is expected to improve the detection rate of fetal heart abnormalities with US.

There have been intelligent studies that focused on the heart rate (63), heart segmentation (64, 65), and congenital heart disease diagnosis (66) of fetus. Yeo et al. have been studying fetal intelligent navigation echocardiography (FINE) for many years. FINE is a novel method for visualization of the standard fetal echocardiography views and application of “intelligent navigation” technology (67, 68). It can obtain nine standard fetal echocardiography views—that is, four chamber, aortic arch, three vessels and trachea, etc—in normal hearts (67) and display abnormal anatomy and doppler flow characteristics (69), and detect complex CHD (70–72). Other researchers have also performed several studies related to FINE (73–77). The results have shown their potential value in fetal heart evaluations and usefulness in the congenital defect screening process, such as double-outlet right ventricle and the D-transposition of large arteries. Moreover, FINE has been integrated into the commercial application 5D Heart (78, 79), which is considered to be a promising tool for cardiac screening and diagnostics in a clinical setting. The exploration of FINE aims to simplify the examination of fetal hearts, reduce operator dependency, and improve the index of suspicion of CHD.

## Discussion

AI is expected to change medical practices in ways we remain unaware of. The latest ultrasound machines are already equipped with intelligent applications. It can realize intelligent measurement based on the standard section obtained by the sonographers. Some ultrasound instruments can realize intelligent imaging of the fetal face. Intelligentized antenatal US could improve work efficiency, provide more consistent and quantitative results, contribute to effective malformation diagnoses. Moreover, AI-based prenatal US promises to improve the quality control of clinical work and the imbalance of medical resources, shortening the training cycle of young doctors.

While CAD in prenatal US is just beginning, the irregular movement of a fetus and the complexity of fetal malformations does pose a significant challenge. To date, most research has been conducted with 2DUS, focused primarily on algorithmic performance rather than clinical utility, with fewer studies exploring intelligent imaging and diagnosis compared to biological measurements.

In terms of diagnosis, comprehensive models need to integrate diagnostic imaging and clinical data. However, in reality, not all cases are easy to diagnosis with only one clear malformation and some malformations may be too subtle. Single abnormal images are too limited for recognition and diagnosis, with data for training being unable to cover all fetus abnormities. Moreover, data used usually come from the same centers, which is insufficient for better robustness making experimental data too limited for generalized clinical applications.

It should be clear that machine learning is a powerful tool for clinical assistance, but it is not realistic to conduct independent diagnosis without the supervision of sonographers. At the same time, because the acquisition of raw data still needs to rely on the sonographers, the sonographer's mistake will also affect the AI diagnosis results. In addition, how to perform intelligent diagnosis integrated with traditional sonographic data is another question that needs to be answered. Furthermore, when designing or applying an algorithm, the false positive or false negative rates need to be considered based on real-world requirements. Finally, the ethical issues related to AI products also need to be considered. All of the above need to be discussed seriously by experts in various fields to pave the way to better health care.

## Conclusion

Multidisciplinary integration is a general trend. There are high expectations of AI applications for innovative healthcare solutions. In this article, we present this emerging technology in the context of US in obstetrics. Despite the advances, there are still many study gaps in this field. AI will continue to be used to optimize prenatal US scans and is expected to provide superior antenatal service in the near future.

## Author Contributions

LC had the idea and designed this review. FH drafted the manuscript and prepared the figures and tables. FH, LC, YW, YX, and YZ revised the paper. All the authors read and agreed to the submission of the final version of the manuscript.

## Funding

This study was supported by the National Natural Science Foundation of China (Grant Nos. 81600258 and 81802540), and the 345 Talent Project of Shengjing Hospital.

## Conflict of Interest

The authors declare that the research was conducted in the absence of any commercial or financial relationships that could be construed as a potential conflict of interest.

## Publisher's Note

All claims expressed in this article are solely those of the authors and do not necessarily represent those of their affiliated organizations, or those of the publisher, the editors and the reviewers. Any product that may be evaluated in this article, or claim that may be made by its manufacturer, is not guaranteed or endorsed by the publisher.

## Abbreviations

AC: Abdominal circumference
AI: Artificial intelligence
CAD: Computer-aided diagnosis
CHD: Congenital heart disease
FFSP: Fetal facial standard plane
FINE: Fetal intelligent navigation echocardiography
FLM: Fetal lung maturity
GA: Gestational age
GS: Gestational sac
HC: Head circumference
NRM: Neonatal respiratory morbidity
NT: Nuchal translucency
ML: Machine learning
2D: Two-dimensional
2DUS: Two-dimensional ultrasound
3D: Three-dimensional
3DUS: Three-dimensional ultrasound
US: Ultrasound.
